# How to overcome violence against Healthcare professionals, reduce medical disputes and ensure patient safety

**DOI:** 10.12669/pjms.311.6446

**Published:** 2015

**Authors:** Hongxing Yu, Zhenglu Hu, Xifan Zhang, Bin Li, Shangcheng Zhou

**Affiliations:** 1 Hongxing Yu, School of Medicine and Health Management, Huazhong University of Science and Technology, Tongji Medical College, No. 13, Road Hangkong, Wuhan, China. Department of Science and Education, Affiliated Dongfeng Hospital, Hubei University of Medicine, Shiyan, China.; 2 Zhenglu Hu, School of Public Health, Sun Yat Sen University, Guangzhou, China.; 3 Xifan Zhang, National Health and Family Planning Commission of the People's Republic of China, Department of Primary Health, Beijing, China.; 4 Bin Li, School of Management, Wuhan University of Technology, Wuhan, China.; 5 Shangcheng Zhou, School of Management, Hubei University of Medicine, Shiyan, China.

**Keywords:** Medical care research, Quality improvement, Violence, Quality of health care, Medical insurance

## Abstract

**Background & Objective:**

In recent years there have been many cases of violence against healthcare professionals (HCPs) in China leading to the death of some doctors as well as nurses by patient’s relatives. Our objective was to identify the causative factors for these violent acts and address these isssues which is vital to ensure patient safety.

**Methods:**

A multidisciplinary research task force was formed to do a root cause analysis of the violent acts against the healthcare professionals. A flowchart was developed to identify the steps in the process and discover the potential links.

**Results:**

There are complex reasons behind the violence against HCPs. However, the main reasons were found to be poor quality of medical services and increased awareness of patients’ rights and their willingness to knock at the doors of courts to seek justice. The feasible counter measures includes stimulating hospital directors to improve patient safety, aligning incentives with quality of service provided in healthcare facilities, monitoring educational quality of HCPs, making necessary changes in medical education programmes besides setting up a reasonable academic promotion mechanism for health professionals based on merit and competence.

**Conclusion:**

Poor quality of medical services, increased awareness among patients about their rights has resulted in increase in medical disputes and at times violence against healthcare professionals. A number of effective measures can be undertaken by the government, hospitals, and medical schools ensuring patient safety. However, it is essential to sensitize the hospital directors to elevate their quality of medical services.

## INTRODUCTION

In China, the incidence of violence against healthcare professionals has significantly increased in recent years. For example, in April 2010, in Shiyan of Hubei Province, 50 relatives of a deceased patient attacked hospital staff, beating and pouring boiling water on the staff.^1^ In more merciless cases, doctors and nurses have been killed by patients or patients’ relatives.^2^ On October 25, 2013, a doctor was stabbed to death by his patient in the outpatient department at the First People's Hospital of Wenling, Zhejiang Province.^3^ On March 23, 2012, a young patient killed a doctor and wounded 3 others in a hospital in Harbin, Heilongjiang Province.^4^ However, besides a few cases of illegal actions,^5^ there are many cases of violence against HCPs in China. One study reported that there are more than one million cases of violence against HCPs every year in China.^1^ One wonder whether these violent acts are inevitable. 

Some studies have analyzed the causes of increased violence against HCPs in China, such commercialization of the medical services system,^6^ poor government investment in the healthcare system,^7^ negative media reports about hospitals and doctors,^8^ catastrophic out-of-pocket medical expenses for families,^9^ and lack of trust in doctors and hospitals.^10^ However, one cannot say with surety whether these are the only factors leading to increasing acts of violence against the HCPs. Our objective in this study was to identify the reasons behind these violent acts and address the causative factors, which is vital to ensure and improve patient safety in China. 

## METHODS

**Fig.1:**
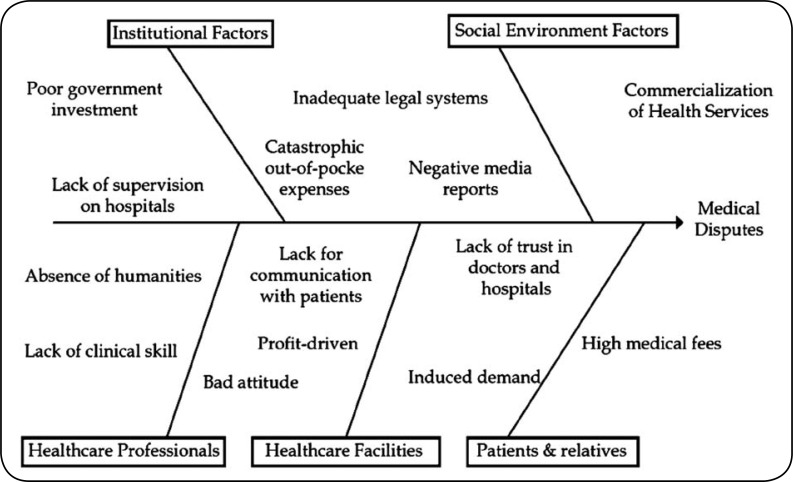
Flowchart depicting the main causes for the incresed violence against HCPs and medical disputes in China

In order to analyze the causative factors for this increasing incidents of violence against HCPs, a multidisciplinary research task force involving hospital managers, hospital clinical department directors, health officials, researchers, lawyers, pharmacists, doctors, and nurses was formed in July 2014. The team did a root cause analysis of the acts of violence. A flowchart was developed to identify the steps in the process and discover the potential links (Fig.1). Two main factors were identified which were contributing to the increasing incidences of violence. One was increased awareness among the patients of their rights besides their legal consciousness while the other was poor quality of medical services. 

Other causes included commercialization of the healthcare system, China embarked on reforms in the early 1980s. The subsequent government funding in the health system was low, at 5% of GDP from 1985 to 2000, but there were few cases of violence against HCPs. From 2009 to 2011, the Chinese government allocated huge financial resources for the healthcare system, with an annual increase of RMB 283.3 billion ($41.42 billion). However, instead of reduction in incidence of violence against healthcare professionals, it has increased. For example, medical malpractice claims in courts increased by 7.6% from 2009 to 2010.^1^

Negative media reports do not directly lead to acts of violence. Once such violent acts have occurred, some individuals do not want to resolve these disputes through legal means but resort to violence or protests because they are encouraged by mass media. This result is the rising number of illegal actions. However, it is difficult to say with certainty that the volume of litigations has actually increased. 

Other factors, such as catastrophic out-of-pocket expenditures on health, lack of trust in doctors and hospitals, inadequacy of legal systems and the absence of any other effective way of addressing the patient’s grievances can also give rise to increase in acts of violence against HCPs. 

## RESULTS

The most important reasons for the increasing acts of violence against the healthcare professionals were increased awareness of patient’s rights, and legal consciousness as well as poor quality of medical services. Patients and their relatives no longer remain silent regarding medical malpractice or medical negligence on the part of doctors or nurses. On the contrary, they want to identify why unexpected results happened and wish to receive adequate explanations besides demanding compensation from hospitals. This has given rise to increasing disputes between the patients and healthcare professionals and healthcare facilities. 

***Lack of quality care and competition among hospitals:*** Public hospitals are dominant because the scope of private medical facilities is extremely low.^11^ Until 2012, the proportion of beds in private hospitals was below 10%.^12^ At the same time, there is also a lack of quality competition among public hospitals. On one hand, there are municipal, district, and community hospitals, and it is impossible for the low-level hospitals to compete with state of the art healthcare facilities; large hospitals compete with small medical facilities for individual patients, which is a negative competition. On the other hand, even though there is competition among some tertiary hospitals, it is based on the hospitals’ scale rather than the quality of medical services because patients are attracted to the hospitals’ keeping in view their equipment, beds, and buildings rather than quality of service.

***Hospital directors have little incentive to improve quality: ***Because the vast majority of public hospitals in China are state owned, multiple government sectors control the hospitals. For example, the financial sector controls hospital inputs, the development and reform commission controls basic construction projects and equipment, and the bureau of price monitors the cost of medical services, medicines and diagnostics. Since all the administrative sectors want to control the hospitals, none of the sectors are able to effectively supervise the hospitals, hence the monitoring of services being provided is not effective. Public hospital directors are appointed by the regional government. The health authorities do not have influence over these appointments, and the directors are not answerable to the health department. The regional governments are unfamiliar with the healthcare industry, making it difficult for them to regulate the directors and the hospitals. At the same time, the directors are more concerned with profits and the hospitals’ popularity in the medical market; therefore, the hospitals’ internal management is focused on productive efficiency rather than on the quality of medical services. As a result, directors often use economic indicators to assess doctors’ job performance, such as inpatient admissions, surgery visits, and outpatient visits, but the directors ignore quality management and medical staff training. Furthermore, the staff do not invest in continuing medical education; at the same time, the medical association has little power to supervise the continuing medical education programme. These factors taken together make it difficult to embark on continuous professional development of the healthcare professionals.

***Health authorities do not efficiently supervise quality:*** The local health bureau is responsible for routine medical administration, including the management of medical professional and institution assessments, blood management, and hospital infection control. Because the health bureaus do not use efficient supervision measures or quality assessment mechanisms, the effect of this regulation is doubtful. For example, the bureaus usually conduct the hospital accreditation programme; however, there are a number of reasons why this programme cannot ensure to improve the quality of hospital services. First, the accreditation standards focus on the hospitals’ scale and productive efficiency rather than the quality of services. Second, many hospitals falsify medical records and statistics to receive higher scores.^13^ Third, the hospitals are not immune to the pitfalls of bureaucracy in the accreditation process. 

***Medical insurance agencies have little influence on quality:*** There are several health insurance programmes in China, including the Urban Employee Basic Medical Insurance, the Urban Resident Basic Medical Insurance, New Rural Cooperative Scheme, and the Government Insurance System.^14^ Because these insurance programmes are managed by several different sectors, including the health bureau, the personnel bureau, and large state-owned enterprises, respectively, it is impossible for all these institutions to use a uniform management system. Meanwhile, because their administrative capacity is low, medical insurance agencies have unequal bargaining power and cannot exert influence over hospitals and doctors. In addition, the main payment methods are fee-for-service and capitation payment systems, which are unreasonable and have led to poor quality in the form of over-provision and over-utilization of services and medicines. Moreover, patients prefer to seek care in better hospitals in other regions, which makes it more difficult for insurance agencies to supervise the quality of medical services. 

***Problems in the medical education system resulting in decreased quality:*** Some research have highlighted problems in the medical education system in China, which include unreasonable curriculum setting, absence of humanities education, and traditional “teacher-centered” teaching methods.^15^ Nevertheless, the two main problems resulting in poor quality of medical services are as follows. 

The total number of medical school in China at present are 644. It includes 146 institutions of higher education for medicine and 498 secondary medical vocational schools. (Source: Chinese Education Statistical Yearbook. 2012, in Chinese). The medical schools have experienced unprecedented growth in admisisons since 1998. Yearly enrolment into these schools increased six-fold from 75,000 in 1998 to almost 450,000 in 2008.^16^ However, government sectors have not increased financial inputs, resulting in a shortage of teaching resources,^17^ which has decreased the quality of education. 

Second, since 2002, graduates have been faced with many difficulties in finding employment.^18^ Meanwhile, most graduates do not want to work in rural or remote areas; as a result, many graduates have joined other industries, such as drug distribution. As noted in the literature, the number of newly employed health staff accounted for only 20%–40% of medical graduates in 2004–2008.^16^

As such due to poor quality, the incidence of medical errors has increased, the length of hospital stays is prolonged, and medical expenses are increased meanwhile, patients’ legal consciousness is also rising. When treatment results in adverse effects, patients or their relatives want to receive compensation from hospitals, causing medical disputes leading to even violent acts.

## DISCUSSION

Since the causative factors leading to increased violence and medical disputes have been identified, we can therefore focus on improving the quality of services to reduce the incidence of such disputes. One study has reported that public hospital directors in China do not have the authority to discipline their employees.^7^ However; hospital directors can manage their hospitals well if they are given powers to appoint and remove the staff based on their performance. They should be able to review the staff performance and give input in development of services and planning. The directors act as the most important persons in hospital development. Therefore, we believe that designing an effective incentive and constraint mechanism to monitor hospital directors would strengthen hospitals’ quality management, train professionals, and improve clinical services. As a result, patient safety would be improved resulting in reduction in medical disputes and acts of violence. How do we encourage the hospital directors to improve the quality of medical services needs to be looked into. It is critical to implement effective and reliable performance assessment tools to evaluate the performance of hospital directors. 

***Medical insurance sectors aligning incentives with hospital quality:*** The Chinese government is integrating its health insurance systems, which means the above-mentioned insurance programmes will be managed by one sector. This reform will not only improve the efficiency of fund utilization but will also encourage hospital directors to improve the quality of medical services because of the increased bargaining power of insurance bodies. In addition, by reforming provider payments, these agencies can indirectly influence the quality of hospital services. For example, the agencies could encourage the director to implement clinical pathways under the combined methods of the global budgeting and DRGs (Diagnosis Related Groups). Because of declines in the use of unnecessary technologies and medicines, patient safety could be improved and medical disputes reduced. Furthermore, medical insurance agencies have more strength than the health authorities to publicly disclose clinical quality indicators, which has the potential to increase medical quality.

***Reforms in medical education system:*** In the short term, it will not be easy for the government to increase its inputs into the medical education system in China. Therefore, the priority should be to conduct a series of strict accreditations for medical schools and eliminate schools with poor quality and standards. To ensure that accreditation is objective and impartial, it is necessary to set up scientific accreditation standards and invite foreign experts to evaluate the performance of all these medical schools. Those institutions which are denied accreditation after inspection should be closed after giving them some time to improve and make up the deficiencies pointed out by the inspection team. The number of enrollments in the medical schools should be based on the number, quality of teachers and other teaching facilities.

## CONCLUSION

With the societal and economic developments in China, medical disputes could still increase because of increased awareness among patients about their rights. A small proportion of these medical disputes are inevitable, but the majority can be prevented. There are many factors responsible for the increase of medical disputes, including the government, patients, and the media. However, the important causes are the poor quality of medical services and the rise in patients’ legal consciousness. Patients claim compensation for losses, and few people resort to violence against healthcare professionals to voice their grievances. It is important to decrease medical disputes to protect the legal interests of both doctors and patients by reinforcing hospital security and protecting the safety and dignity of medical staff, as well as settling medical disputes under the law. However, reforming the legal and judicial system in China would be costly and could take years to complete. Hence to reduce medical disputes, the key measure is to gradually improve the quality of medical services and prevent avoidable disputes. There are many actions which can be taken by the government, hospitals, and medical schools, but the most critical is to encourage public hospital directors to enhance and improve the quality of medical services.
